# Tibial Component Malalignment in Total Ankle Arthroplasty

**DOI:** 10.3390/medicina62101920

**Published:** 2026-10-05

**Authors:** Virginia Cinelli, Gloria Assegbede, Antonio Mascio, Chiara Comisi, Federico Moretti, Vincenzo Mancino, Tommaso Greco, Giulio Maccauro, Carlo Perisano

**Affiliations:** 1 Department of Orthopedics and Geriatric Sciences, Catholic University of the Sacred Heart, 00168 Rome, Italy; 2 Department of Orthopedics, Ageing and Rheumatological Sciences, Fondazione Policlinico Universitario Agostino Gemelli IRCCS, 00168 Rome, Italy; 3 Department of Life Sciences, Health, and Healthcare Professions, Link Campus University, 00165 Rome, Italy

**Keywords:** total ankle arthroplasty, tibial component, malalignment

## Abstract

*Background*: Total ankle replacement (TAR) has emerged as a valuable alternative to ankle arthrodesis for the treatment of end-stage ankle osteoarthritis, offering pain relief while preserving joint motion and improving quality of life. Despite advances in implant design and surgical techniques, complications remain common, and tibial component malalignment has been increasingly recognized as a major contributor to implant failure and revision surgery. *Purpose*: To provide a comprehensive overview of the current evidence regarding tibial component malalignment in TAR, focusing on its etiology, radiographic assessment, biomechanical implications, clinical consequences, and available treatment strategies. *Methods*: A narrative review of the literature was conducted using PubMed, Scopus, and Web of Science. English-language studies published up to 2026 investigating tibial component positioning, malalignment, associated complications, and revision strategies in TAR were included and qualitatively analyzed. *Results*: Tibial component malalignment is a multifactorial condition influenced by preoperative deformities, patient-specific anatomy, surgical technique, instrumentation, and surgeon experience. Malposition in the coronal, sagittal, or axial planes alters ankle biomechanics, resulting in abnormal load distribution, altered kinematics, polyethylene wear, instability, and increased stress at the bone–implant interface. These changes may lead to pain, aseptic loosening, subsidence, osteolysis, and ultimately prosthetic failure. Weight-bearing radiographs remain the standard imaging modality for postoperative assessment, while weight-bearing computed tomography provides complementary three-dimensional evaluation, particularly in complex cases. Management should be individualized according to implant stability, degree of deformity, and symptom severity, ranging from supramalleolar osteotomy and isolated polyethylene insert exchange to partial or complete revision arthroplasty, including the use of custom implants in cases of substantial bone loss. *Conclusions*: Tibial component malalignment represents a critical determinant of functional outcomes and implant survivorship following TAR. Early recognition through standardized imaging and a thorough understanding of its biomechanical and clinical implications are essential to optimize treatment strategies, minimize revision rates, and improve long-term outcomes after total ankle replacement.

## 1. Introduction

Ankle osteoarthritis (OA), characterized by progressive degeneration of the tibiotalar joint, is a debilitating condition that leads to significant pain and a gradual loss of patient autonomy and quality of life [1]. Traditionally, tibiotalar arthrodesis has been considered the gold standard for end-stage disease, particularly in younger and active patients, due to its reliability and durable long-term outcomes. However, total ankle replacement (TAR) has increasingly gained acceptance as a valuable alternative, offering the advantage of preserving joint motion and improving overall quality of life [2]. Although revision rates remain relatively high, advances in implant design have led to encouraging mid-term clinical outcomes [3,4]. Over the past 15 years, growing evidence has supported the role of TAR as a valid alternative to ankle arthrodesis, allowing restoration of joint function, stability, and alignment, with clear benefits in terms of gait and patient independence [5].

Despite these advances, TAR is still a technically demanding procedure and continues to challenge orthopedic surgeons, largely due to the persistence of complications [6]. Over the years, substantial improvements have been made in implant design, fixation methods, and bearing materials, leading to enhanced implant survivorship and improved functional outcomes. Despite these advances, implant positioning remains a critical determinant of procedural success, and component malalignment—particularly of the tibial component—continues to be one of the leading causes of mechanical failure and, consequently, the need for revision surgery for complications.

Although neutral alignment is commonly considered the reference target in TAA, it should not necessarily be regarded as a universal goal for every patient. Unlike total knee arthroplasty, where personalized alignment strategies have been extensively investigated [7], no universally accepted patient-specific alignment philosophy has yet been established in TAA. However, individualized alignment based on native tibial and hindfoot anatomy, preoperative deformity, soft-tissue balance, and implant design is increasingly considered. Therefore, neutral alignment should be regarded as a radiographic reference rather than an absolute target, with the ultimate goal of restoring a balanced and mechanically favorable ankle–hindfoot construct.

Notwithstanding these technological improvements, the success of TAR still relies heavily on accurate implant positioning. In particular, malalignment of the tibial component has emerged as a critical factor influencing outcomes and is recognized as one of the leading causes of early failure. Even small deviations in the coronal, sagittal, or axial planes can alter load distribution across the joint, leading to abnormal stress concentration, instability, and accelerated wear of the polyethylene insert. There is no universally accepted definition of tibial component malalignment, which represents a major limitation when comparing outcomes, as clear threshold values are lacking. Most studies define it as a deviation of the implant, in the coronal, sagittal, and axial planes, from the planned alignment [8,9,10]. Coronal deviations greater than 5–10 degrees are generally considered clinically relevant, while deviations exceeding 15 degrees are regarded as severe. In the sagittal plane, excessive anterior or posterior slope can alter ankle kinematics and component loading, whereas rotational malalignment remains more difficult to quantify due to the absence of standardized radiographic reference points.

Clinically, tibial component malalignment can result in persistent pain, reduced function, and mechanical complications such as impingement and instability. Over time, these alterations may progress to aseptic loosening and loss of implant fixation, often necessitating revision surgery. Coronal plane malalignment, in varus or valgus, has been linked to eccentric loading patterns that compromise implant survival and negatively impact clinical outcomes. Similarly, sagittal plane malpositioning may disrupt joint kinematics, further affecting stability and functional performance.

Given these considerations, tibial component malalignment plays a pivotal role in the development of complications following TAR. A deeper understanding of its biomechanical and clinical consequences is essential to refine surgical techniques, minimize failure rates, and ultimately improve patient outcomes. The aim of the present study is to investigate the complications associated with tibial component malalignment in total ankle replacement, with particular attention to its effects on clinical outcomes and implant survival.

This article is a narrative literature review aimed at providing a clinically oriented synthesis of the available evidence on tibial component malalignment in total ankle replacement; it was not designed as a systematic review and therefore does not include protocol registration or a formal risk-of-bias assessment.

A narrative literature review on tibial component malalignment in TAR was conducted using the principal medical databases, including PubMed, Scopus, and Web of Science. The search included articles published in English up to 2026 using combinations of the keywords “total ankle replacement”, “ankle arthroplasty”, “tibial component”, “malalignment”, “implant positioning”, and “complications”. Studies focusing on the biomechanical impact, radiographic assessment, surgical techniques, and clinical consequences of tibial component malalignment were considered. Particular attention was given to investigations reporting complications such as aseptic loosening, implant failure, polyethylene wear, and the need for revision surgery. Relevant articles were selected based on their methodological quality and scientific relevance and were qualitatively analyzed to provide a comprehensive overview of the current evidence regarding the impact of tibial malalignment on TAR outcomes.

## 2. Etiology and Risk Factors of Tibial Component Malalignment

Tibial component malalignment in TAR is a multifactorial phenomenon arising from the interaction of patient-related factors, pre-existing deformities, intraoperative technical challenges, and implant-related limitations. It is usually not the result of a single technical error but rather the final expression of a complex interplay between anatomical, biomechanical, and surgical variables.

Preoperative deformity represents one of the most important determinants of tibial component positioning. Patients with severe varus or valgus ankle osteoarthritis often present with complex three-dimensional deformities involving the distal tibia, hindfoot, and surrounding soft tissues. In these cases, restoration of a neutral mechanical axis may be challenging, and residual malalignment may persist despite accurate intraoperative technique [8,9]. Post-traumatic ankle osteoarthritis is particularly demanding, as previous fractures, malunions, and bone loss may obscure anatomical landmarks and alter the relationship between the mechanical and anatomical axes [10,11].

Cook et al. present a treatment algorithm for TAR in patients with end-stage ankle arthritis associated with varus or valgus deformities. The decision-making process is based on the tibiotalar angle (TTA), joint congruency, and hindfoot alignment angle (HAA). If the hindfoot is neutral, TAA can usually be performed in a single stage using coupled tibial and talar cuts. When hindfoot deformity contributes to the malalignment, additional soft-tissue balancing or staged corrective procedures (osteotomies or hindfoot fusions) are recommended before TAA. Through 12 representative cases, the authors show that severe coronal plane deformities are not necessarily a contraindication to ankle replacement when meticulous preoperative assessment and individualized correction strategies are employed [12].

Supramalleolar osteotomy (SMO) has been proposed as an adjunct to TAR in patients with severe distal tibial deformities to restore a more physiological mechanical axis and facilitate ankle realignment. However, comparative evidence suggests that SMO does not provide superior pain relief or functional outcomes compared with TAR alone [13]. Its role is therefore primarily biomechanical, and it should be reserved for selected cases with severe extra-articular deformities in which restoration of a neutral weight-bearing axis cannot be achieved through prosthetic implantation alone.

Intraoperative factors also play a crucial role in determining final implant alignment. Accurate tibial component positioning depends on correct identification of anatomical landmarks, precise execution of bone resections, and adequate soft-tissue balancing. Errors in tibial cutting angle or reference alignment may result in coronal or sagittal malpositioning, with subsequent alteration of load distribution across the prosthetic joint [14].

Instrumentation systems significantly influence the accuracy of tibial alignment. Extramedullary guides are widely used but may be less reliable in cases of severe deformity or obesity. Intramedullary referencing systems may improve sagittal alignment accuracy; however, their use can be limited by anatomical variations or previous intramedullary pathology. Patient-specific instrumentation and computer-assisted navigation have been introduced to improve reproducibility of component positioning, although their clinical superiority over conventional techniques remains debated in the literature [15,16,17,18].

The surgeon learning curve is another factor that can affect component alignment, especially when a new implant system or a new technique such as patient-specific instrumentation is introduced. Early series suggest that radiographic consistency may improve with increasing familiarity, whereas any effect on complication rates appears less clear [19].

Overall, tibial component malalignment should be considered the result of multiple interacting factors, including patient anatomy, deformity severity, surgical technique, and implant-related constraints. Understanding these contributors is essential to optimize preoperative planning, improve intraoperative accuracy, and potentially reduce the risk of mechanical failure in total ankle replacement.

## 3. Radiographic Assessment

Radiographic assessment plays a central role in evaluating tibial component positioning in TAR, as implant alignment is directly associated with functional outcomes, complication rates, and implant survival. Although weight-bearing radiographs (WBXR) remain the standard imaging modality, their two-dimensional nature limits the accurate assessment of complex multiplanar alignment.

Accurate radiographic assessment of prosthetic positioning relies on angular and linear measurements obtained on weight-bearing anteroposterior (AP) and lateral radiographs [20,21]. In the OARS cohort, angular measurements showed higher reliability than linear measurements, with a coefficient of variation of ≤8% for angles versus ≤17.7% for linear measures [20]. Nevertheless, conventional radiographs are limited in assessing complex multiplanar alignment, and weight-bearing CT-based analyses have shown good-to-excellent reproducibility and only moderate agreement with standard weight-bearing radiographic measurements for several parameters [22,23,24].

The primary parameters for assessing tibial component alignment are the α and β angles, which ideally approximate 90° with a tolerance of ±3° [14,17]. These prosthetic angles can be interpreted in relation to native anatomical reference angles (Medial Distal Tibial Angle (MDTA) and the Anatomic Sagittal Distal Tibial Angle (aSDTA)).

### 3.1. Anteroposterior (AP) View: (Figure 1)

**α angle or tibial anterior surface angle (TAS)**: defined by the intersection between the tibial anatomical axis (TAX) and a line parallel to the anterior surface of the tibial component.

**Tibiotalar surface angle (TSA)**: formed by the intersection of TAX and a line parallel to the anterior surface of the talar component.

**Hindfoot alignment angle (HFA)**: defined by the intersection between TAX and the longitudinal anatomical axis of the calcaneus (CAX), providing an overall assessment of hindfoot alignment.

### 3.2. Sagittal (Lateral) View: (Figure 2 and Figure 3)

**β angle**: defined by the intersection between TAX and a line perpendicular to the medial surface of the tibial component.

**γ angle**: defined by the intersection between a line perpendicular to the medial aspect of the talar component and a line connecting the center of the talar head to the posterior aspect of the talar component, reflecting talar component orientation.

**Tibiotalar ratio (TTR)**: evaluates the anteroposterior position of the talus relative to the tibia. It is measured along the talar longitudinal axis (TALAX), using three reference points:

Point A: posterior aspect of the talar component.

Point B: intersection between TAX and TALAX.

Point C: intersection between TALAX and the talar head.

The TTR is calculated as the ratio AB/AC, expressed as a percentage.

The TTR is a sagittal alignment measurement for the ankle, with a normal value typically between 27% and 42%. Values below this range indicate an anterior shift of the talus, while values above it signify posterior displacement.

**Anteroposterior offset ratio (AP Offset Ratio)**: evaluates the anteroposterior position of the talar component relative to the tibial axis. It is measured on a weight-bearing lateral radiograph using two reference measurements:

X: perpendicular distance between the longitudinal tibial axis and a line passing through the center of the talar component, parallel to the tibial axis.

Y: anteroposterior length of the tibial tray/component.

The AP Offset Ratio is calculated as the ratio X/Y, with the result expressed as a decimal or percentage.

The AP Offset Ratio is a sagittal alignment measurement of the ankle following total ankle replacement. A value of 0 represents neutral alignment, indicating that the center of the talar component is aligned with the longitudinal tibial axis. Positive values indicate anterior translation of the talar component, whereas negative values indicate posterior translation. Values close to zero are generally considered desirable, although the acceptable range may vary according to implant design and measurement technique. A commonly used reference range is approximately −0.05 to +0.05 (−5% to +5%), corresponding to a near-neutral position.

Overall, these parameters provide a comprehensive multiplanar assessment of implant positioning, integrating tibial alignment (α, β), talar orientation (TSA,γ), and global ankle–hindfoot relationships (HFA, TTR).

Regarding the classification of coronal malalignment, the literature defines neutral alignment as deviation less than 5° of varus or valgus, moderate malalignment when between 5° and 15°, and severe when exceeding 15° [14,25,26].

The sagittal position of the talar component, assessed using the AP Offset Ratio, holds particular clinical importance. In a study of 317 consecutive patients, Barg et al. demonstrated that patients with an AP offset ratio equal to zero exhibited significantly lower pain levels compared to those with a positive ratio (*p* = 0.001) or negative ratio (*p* = 0.017), in addition to higher American Orthopaedic Foot and Ankle Society scores (AOFAS score) and greater range of motion [9]. The aSDTA, (normal value 80–85°), reflects sagittal plane orientation and demonstrates a strong correlation with talar positioning (r = 0.81), with surgical correction reducing the risk of prosthetic subluxation by approximately 0.6–1.1 mm per degree of angular adjustment [27].

The accuracy of tibial component positioning depends significantly on the surgical technique employed. Comparison between extramedullary and intramedullary reference systems revealed that the latter provides greater precision in the sagittal plane (2.5° ± 1.8° vs. 4.1° ± 2.9°, p0.001), while in the coronal plane both techniques show comparable results (1.4° ± 1.1° vs. 1.5° ± 1.4°) [14]. The use of patient-specific instrumentation represents a further technological evolution aimed at improving positioning accuracy [17].

The consequences of tibial component malalignment manifest through a cascade of pathological events including asymmetric load distribution, accelerated polyethylene wear, aseptic loosening, and ultimately implant failure. Radiographic signs of complications include periprosthetic radiolucent lines, found in 31–39% of cases at the tibial level and 3% at the talar level, component subsidence (8–24%), periprosthetic osteolysis (34%), and periprosthetic fractures (11%) [28,29,30].

Criteria for diagnosing loosening include progressive radiolucent lines greater than 2 mm, change in component position compared to previous radiographs, subsidence greater than 5 mm, and progressive osteolysis [29,31]. Computed tomography (CT) has proven superior to standard radiographs in detecting osteolytic lesions, identifying a greater number of lesions of larger dimensions, and is therefore recommended in patients with suspicious radiolucencies, persistent pain, or during preoperative evaluation for revision (Table 1).

Radiographic assessment of TAR positioning is a critical aspect in evaluating clinical outcomes and detecting early complications. WBXR have traditionally been considered the gold standard; however, they are inherently limited by their two-dimensional nature, projection errors, and variability related to patient positioning. In this context, weight-bearing cone beam computed tomography (WBCT) has emerged as an advanced imaging modality capable of providing a more accurate three-dimensional evaluation of prosthetic alignment.

Recent evidence has demonstrated that WBCT offers excellent intra- and interobserver reliability (ICC 0.85–0.95), supporting its robustness in assessing key alignment parameters. However, when compared with conventional radiographs, only moderate agreement has been observed for several commonly used measurements (e.g., α angle, β angle, tibiotalar surface angle, and tibiotalar ratio), with good agreement limited to the talar component angle (γ). Notably, poor or absent agreement has been reported for hindfoot alignment and axial rotation, highlighting that WBCT and WBXR should not be considered interchangeable, but rather complementary modalities [23].

From a clinical perspective, the ability of WBCT to generate three-dimensional, weight-bearing models allows for a more comprehensive understanding of ankle and hindfoot biomechanics, particularly in the presence of complex or multiplanar deformities that may be underestimated on standard radiographs [32]. This capability is especially relevant in the evaluation of prosthetic malalignment, including tibial component positioning [23]. Furthermore, WBCT may enhance preoperative planning, postoperative assessment, and the development of patient-specific technologies [32]. Despite these advantages, the widespread adoption of WBCT remains limited by factors such as cost, availability, and the need for dedicated software for image processing [33]. In addition, metal artifacts and challenges in image processing/segmentation may affect measurement accuracy in some cases [34].

**Biomechanical Consequences of Tibial Malalignment**

Tibial component malalignment in TAR refers to improper implant positioning relative to the tibial axis in the coronal, sagittal, or axial planes. This condition alters joint biomechanics, leading to abnormal load distribution, impaired kinematics, and increased stress at the bone–implant interface, with a higher risk of loosening, subsidence, and poor clinical outcomes [15,35,36,37].

Cadaveric and simulation studies consistently support that even small deviations in component positioning alter load transfer patterns at both the joint and bone–implant interface level, rather than producing only geometric misalignment [15,35,38].

Coronal malalignment (varus/valgus) affects foot mechanics and plantar pressures, while sagittal errors, particularly excessive posterior slope, compromise joint alignment and soft tissue balance [35].

In a cadaveric gait simulation study, Buckner et al. demonstrated that coronal malalignment of the tibial insert significantly alters distal foot loading patterns, producing measurable changes in plantar pressure distribution and midfoot/hindfoot bone mechanics under physiologic loading conditions [35]. This provides direct experimental evidence that coronal malalignment affects not only implant mechanics but also downstream skeletal loading.

Similarly, Saito et al. showed that tibial component positioning has a direct effect on postoperative ankle kinematics during simulated gait, with malalignment leading to altered tibiotalar motion patterns and deviations from physiological ankle movement trajectories [36]. These kinematic changes may represent an early functional manifestation of malalignment before radiographic failure becomes evident.

From a biomechanical perspective, sagittal plane malalignment is particularly relevant because it is closely linked to altered tibiotalar axis alignment. Cho et al. demonstrated that failure to restore sagittal alignment correlates with mismatch between tibial axis and talar component positioning, resulting in persistent malalignment of the reconstructed joint even after implantation [37].

In another study, Van Hoogstraten et al. demonstrated that malalignment of the total ankle replacement leads to a substantial increase in peak contact stresses at the bone–implant interface. In the neutral model, peak contact pressure at the tibial interface was 98.4 MPa, increasing up to 177 MPa in 10° valgus malalignment [15]. Shear stresses also increased significantly, indicating that malalignment affects both compressive and tangential loading components.

Importantly, stress concentration was not uniform but localized predominantly at the edges of the implant–bone interface. This edge-loading phenomenon is considered biomechanically relevant, as trabecular bone is particularly sensitive to focal overload and may fail under repetitive stress conditions even below ultimate strength thresholds [15,39].

The same study also highlighted that valgus and posterior malalignment produce the most pronounced increases in contact stress, suggesting that not all planes of malalignment have equivalent biomechanical consequences [15].

Rotational malalignment can further disrupt normal ankle motion. However, compared to coronal and sagittal plane malalignment, the biomechanical evidence regarding rotational errors remains less robust and is mainly based on kinematic inference rather than direct interface stress measurement [36,40,41,42].

Axial rotational alignment remains less standardized than coronal and sagittal alignment. In a study of 58 patients undergoing mobile-bearing TAA, Hintermann et al. assessed the relative axial rotation between the tibial and talar components intraoperatively and in 48 patients using weight-bearing CT at a mean follow-up of 6.3 years. The mean relative talar rotation was 1.7° toward internal rotation intraoperatively and 1.4° postoperatively, with a wide individual range of rotation. Notably, preoperative sagittal alignment of the distal tibia correlated with intraoperative axial rotation. These findings suggest that axial positioning may be strongly influenced by patient-specific anatomy and that a single universal rotational target may not be appropriate. The wide interindividual variability also supports the use of three-dimensional imaging, particularly weight-bearing CT, when axial malpositioning is suspected after TAA [41].

A cadaveric study investigated ankle and hindfoot kinematics before and after TAR. Results showed no significant differences in sagittal or coronal plane motion between native and prosthetic conditions; however, a significant increase in internal talar rotation in the transverse plane was observed after TAR. This alteration was strongly correlated with the medial–lateral position of the tibial component (β = 1.86°/mm, R^2^ = 0.72, *p* = 0.008). These findings suggest that tibial component positioning plays a key role in postoperative ankle kinematics, particularly in the transverse plane [36].

Achieving neutral alignment—ideally positioning the tibial component perpendicular to the tibial axis—is therefore essential to optimize function and implant survival [36].

Overall, the combination of experimental gait studies, kinematic analyses, and finite element modeling consistently indicates that tibial component malalignment produces a cascade of biomechanical alterations involving:

- Altered distal foot loading patterns [35];
- Abnormal ankle kinematics [36];
- Persistent axis mismatch in sagittal alignment [37];
- Increased bone–implant interface stress and edge loading [15].

These mechanisms likely act synergistically, explaining why even relatively small deviations from neutral alignment can contribute to clinically relevant complications such as loosening and subsidence.

The biomechanical effects of tibial component malalignment may vary according to prosthetic design. Fixed-bearing systems constrain the polyethylene insert to the tibial component, whereas mobile-bearing systems allow a degree of insert translation and rotation, potentially modifying load transmission and joint kinematics. However, current clinical evidence does not demonstrate significant differences in overall, major, or minor complication rates between FB and MB total ankle prostheses. Therefore, although implant design may influence the biomechanical response to malalignment, implant-specific tolerance thresholds have not yet been established, and the clinical significance of tibial malalignment should be interpreted in the context of prosthesis design and other patient- and surgery-related factors [43].

## 4. Clinical Consequences

Variations in implant positioning tend to influence pain, function, implant stability, and ultimately prosthetic survivorship.

Pain is typically the first symptom reported by these patients. It is often activity-related and not always easily localized. In some cases, it remains relatively mild for a period of time but tends to worsen as mechanical overload progresses, particularly in the presence of impingement, polyethylene wear, or early loosening. Residual coronal varus alignment is a common finding and has been associated with eccentric loading of the components and increased interface stresses, which in turn correlate with poorer functional outcomes [9].

From a practical standpoint, these patients often present with reduced walking tolerance and difficulty on uneven terrain, as well as a sensation of instability, even when standard postoperative radiographs appear acceptable. Tibial component malposition strongly influences ankle kinematics and the way forces are transmitted through the hindfoot, helping to explain the functional limitations observed in daily activities [9,44].

Instability represents another observable pattern, particularly in cases where malalignment is associated with progressive deformity or polyethylene insert wear. Residual varus or valgus alignment alters ligament tension and compromises the balance of the ankle–hindfoot complex. In revision cohorts, instability is repeatedly reported among the most frequent causes of reoperation, often alongside malposition and polyethylene wear [45].

Overall, tibial component malalignment should be understood as a mechanical problem that progressively influences symptoms, function, and implant survivorship, ultimately leading in many cases to prosthetic failure.

Also, postoperative rehabilitation is an important component of functional recovery after TAA. It aims to restore ankle range of motion, progressively regain weight bearing, improve muscle strength, and optimize gait and balance. However, rehabilitation protocols vary considerably, and no standardized evidence-based regimen has been established. A systematic review of 84 studies reported substantial protocol heterogeneity and insufficient evidence to identify a superior strategy. Therefore, rehabilitation should be individualized according to implant stability, soft-tissue healing, concomitant procedures, and patient-specific factors [46].

## 5. Treatment Options

### 5.1. Supramalleolar Osteotomy

SMO has been described as a potential surgical strategy for correcting varus malposition of the tibial component in selected patients with a stable implant. It is primarily indicated in patients with varus malalignment of the tibial component associated with persistent pain, in the presence of a stable implant without signs of loosening. It is also indicated in cases of distal tibial deformity that cannot be corrected by isolated polyethylene insert exchange. As a strictly extra-articular procedure, it preserves the integrity of the prosthetic joint, thereby avoiding the need for implant revision [47].

In a series of 22 patients with varus malposition of the tibial component, a significant improvement in the anterior distal tibial angle was observed (from 85.2° ± 2.5° to 91.4° ± 2.9°; *p* < 0.0001), along with an increase in the AOFAS score (from 46 ± 14 to 66 ± 16; *p* < 0.0001) and a reduction in pain as measured by the VAS scale (from 5.8 ± 1.9 to 3.3 ± 2.4; *p* < 0.001). In terms of patient satisfaction, 68% of patients reported being very satisfied, 18% moderately satisfied, and 14% not satisfied [47].

### 5.2. Surgical Management of Periprosthetic Cysts

Periprosthetic cysts and osteolytic lesions are among the most common long-term complications following total ankle arthroplasty and represent a major cause of progressive bone loss and subsequent implant failure. Their pathogenesis is multifactorial and involves biological reactions to polyethylene wear particles, stress shielding, micromotion at the bone–implant interface, and altered load distribution across the prosthetic components. Although many cystic lesions remain asymptomatic in the early stages and are detected during routine radiographic follow-up, progressive osteolysis may eventually lead to pain, implant instability, component loosening, and the need for revision surgery. For this reason, regular surveillance with weight-bearing radiographs and, when indicated, computed tomography is essential to assess lesion size, progression, and the integrity of the surrounding bone stock. Surgical treatment is generally recommended for symptomatic patients or for lesions demonstrating radiographic progression. The most commonly adopted strategy consists of cyst curettage combined with defect filling using autologous bone graft, allograft, or synthetic bone substitutes. In cases associated with implant loosening or significant component malposition, cyst treatment should be combined with partial or complete prosthetic revision. Recent studies have shown that early intervention may prevent further bone loss and contribute to maintaining implant survivorship over time [48,49]. In addition, a growing understanding of the biological mechanisms underlying periprosthetic osteolysis has led to the development of new preventive and reconstructive strategies, which have been extensively discussed in recent reviews on complications of total ankle arthroplasty [50].

### 5.3. Prosthesis Revision

Revision surgery remains the definitive treatment when tibial component malalignment is associated with implant loosening, progressive subsidence, extensive osteolysis, polyethylene wear, or persistent symptoms that cannot be addressed through joint-preserving procedures. The surgical strategy depends on the underlying cause and severity of failure, ranging from isolated polyethylene insert exchange in selected cases with well-fixed components to partial or complete component revision. In more complex situations characterized by severe bone loss or substantial deformity, customized implants may be required to restore alignment and achieve stable fixation. Careful preoperative evaluation, including advanced imaging when indicated, is essential to identify the mechanism of failure and guide the most appropriate revision strategy. Several contemporary studies have demonstrated satisfactory clinical and radiographic outcomes following revision TAA, with significant improvements in pain relief, functional scores, and implant survivorship at mid-term follow-up [51]. Furthermore, recent evidence suggests that, whenever technically feasible, revision arthroplasty may provide superior functional outcomes compared with salvage procedures that sacrifice ankle motion [51,52].

### 5.4. Tibiotalocalcaneal Arthrodesis as a Salvage Procedure

Ankle arthrodesis, either isolated tibiotalar fusion or tibiotalocalcaneal arthrodesis, remains the primary salvage procedure when revision total ankle arthroplasty is not feasible or is associated with an unacceptably high risk of failure. Common indications include extensive periprosthetic bone loss, deep infection, severe soft-tissue compromise, massive component loosening, multiple failed revision procedures, and patients with limited reconstructive options. Although arthrodesis inevitably results in the loss of ankle motion, it remains a reliable method for achieving pain relief and restoring a stable, plantigrade foot capable of supporting functional ambulation. The choice of fixation technique depends on the extent of bone loss and residual bone quality and may include internal fixation with screws or plates, retrograde tibiotalocalcaneal intramedullary nailing, or the use of structural bone grafts and bulk allografts in cases of substantial osseous defects [53]. Advances in fixation methods, biological augmentation, and reconstructive techniques have significantly improved fusion rates and reduced the incidence of nonunion. Nevertheless, arthrodesis continues to be associated with greater morbidity and functional limitations compared with successful revision arthroplasty [54]. Despite these drawbacks, current evidence supports arthrodesis as a dependable and effective salvage strategy for complex failures of total ankle replacement, providing satisfactory clinical outcomes even in the most challenging scenarios [55,56,57].

### 5.5. Strengths and Limitations

The main limitation of this work is its narrative design, which is inherently more vulnerable to selection bias than a systematic review and does not allow quantitative pooling of outcomes or formal assessment of heterogeneity. In addition, the available literature on tibial component malalignment after TAR is characterized by heterogeneous definitions, measurement methods (2D WBXR vs. 3D WBCT), implant designs, and outcome reporting, limiting direct comparison across studies and preventing firm threshold recommendations for “clinically relevant” malalignment. Finally, we limited inclusion to English-language articles and to the databases searched, which may have resulted in omission of relevant evidence. The main strengths of this review are the focused scope on tibial component malalignment as a modifiable driver of TAR failure mechanisms, the integration of clinical, imaging, and biomechanical evidence into a pragmatic framework, and the emphasis on standardized postoperative assessment and individualized treatment pathways aligned with implant fixation status and deformity magnitude.

## 6. Conclusions

Tibial component malalignment in total ankle replacement represents a clinically relevant mechanical problem that may compromise ankle biomechanics, functional outcomes, and implant longevity. Accurate assessment through standardized radiographic parameters is crucial for early detection of malposition and its sequelae. While mild deviations may be tolerated, persistent malalignment can initiate a cascade of abnormal load distribution, altered kinematics, polyethylene wear, loosening, and eventual prosthetic failure. Management should therefore be individualized according to the severity of deformity, implant stability, and patient symptoms, ranging from corrective extra-articular procedures to revision arthroplasty. Ultimately, achieving and maintaining appropriate alignment remains one of the key determinants of successful long-term outcomes after total ankle replacement.

## Figures and Tables

**Figure 1:**
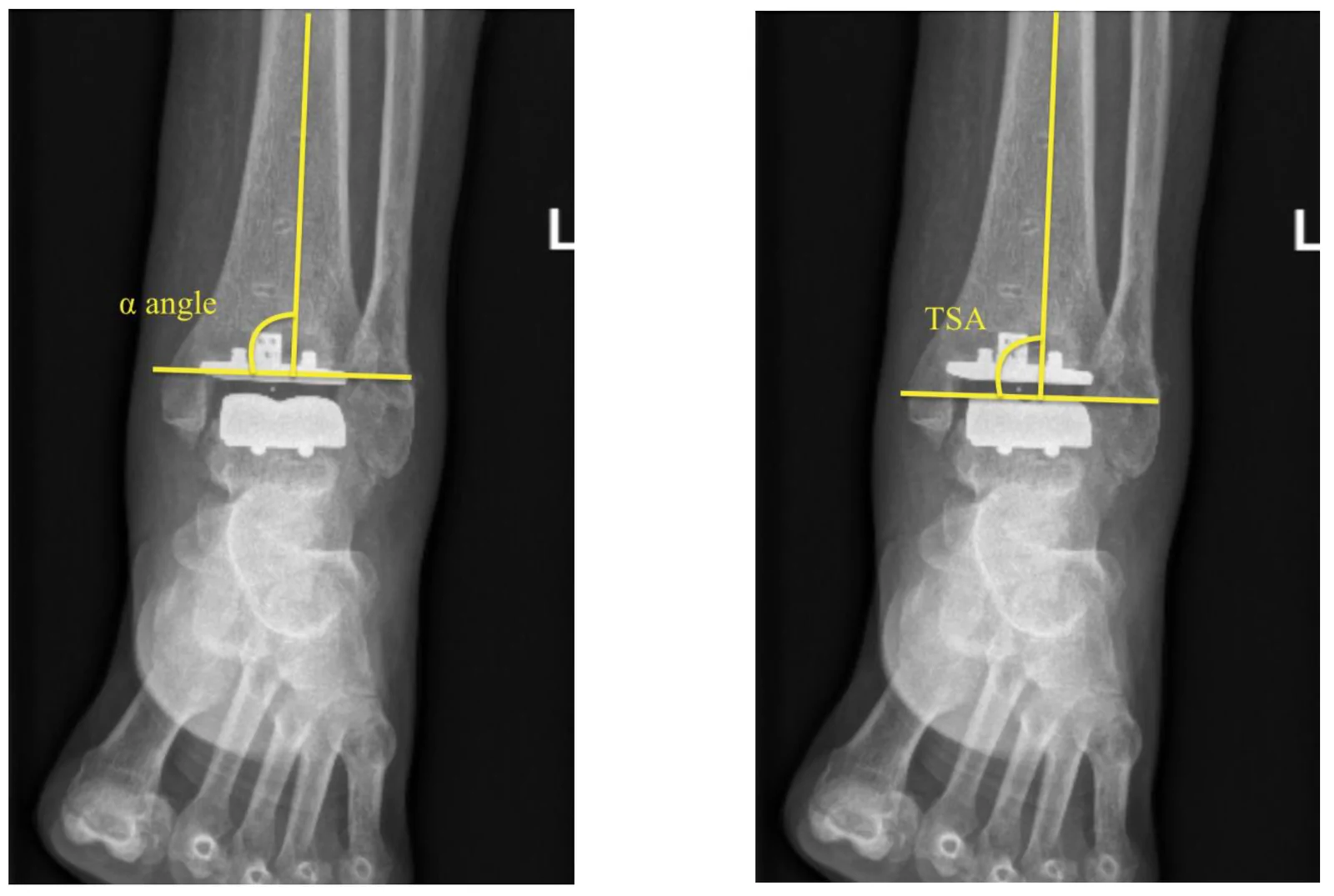
Anteroposterior ankle radiograph. On the left—**α angle**, defined as the angle between the tibial anatomical axis (TAX) and a line parallel to the anterior surface of the tibial component. On the right -**Tibiotalar surface angle (TSA)**, defined as the angle between the TAX and a line parallel to the anterior surface of the talar component.

**Figure 2:**
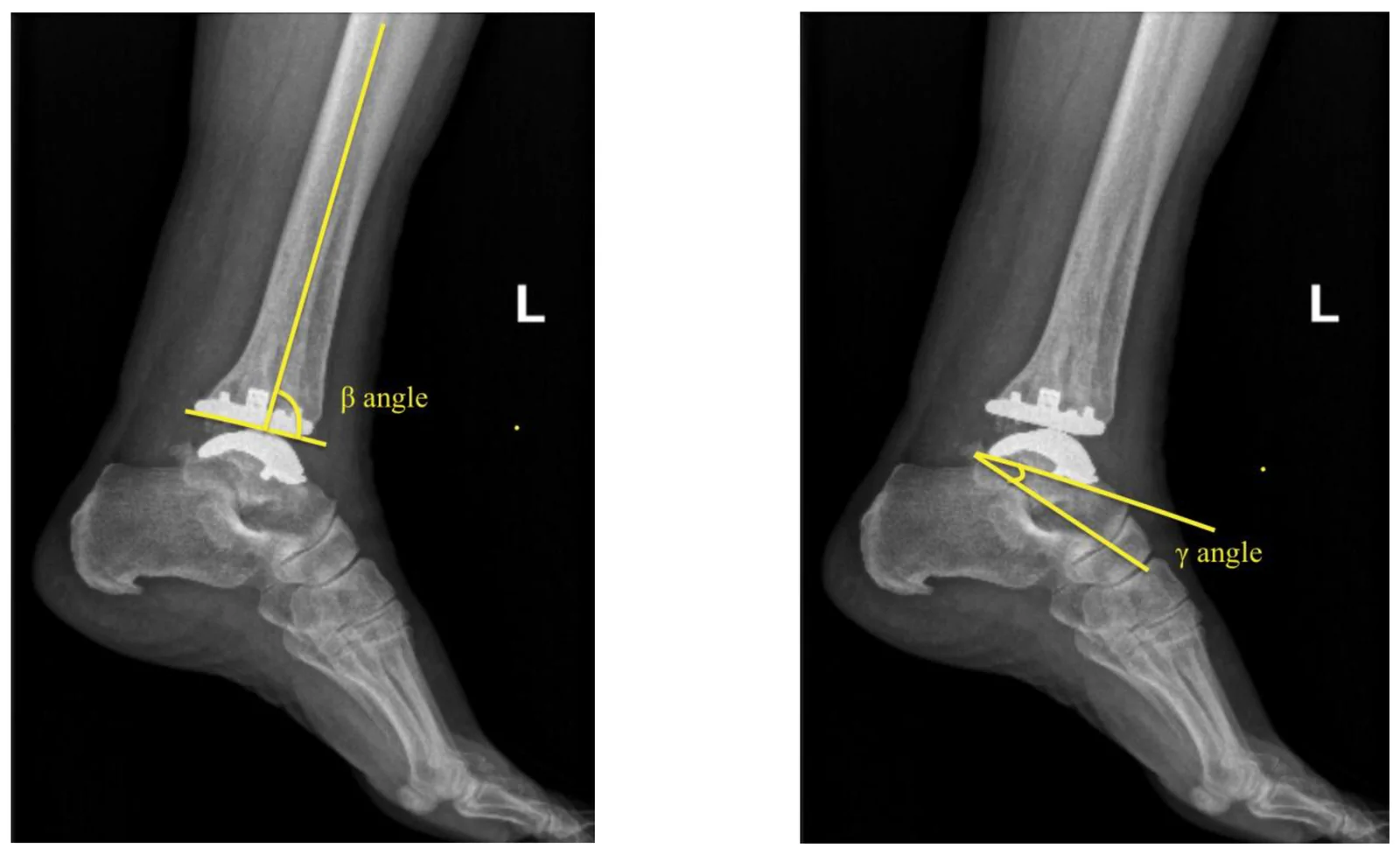
Lateral ankle radiograph. On the left—**β angle**, defined as the angle between the tibial anatomical axis (TAX) and a line perpendicular to the medial surface of the tibial component. On the right **γ angle**, defined as the angle between a line perpendicular to the medial aspect of the talar component and a line connecting the center of the talar head to the posterior aspect of the talar component, reflecting talar component orientation.

**Figure 3:**
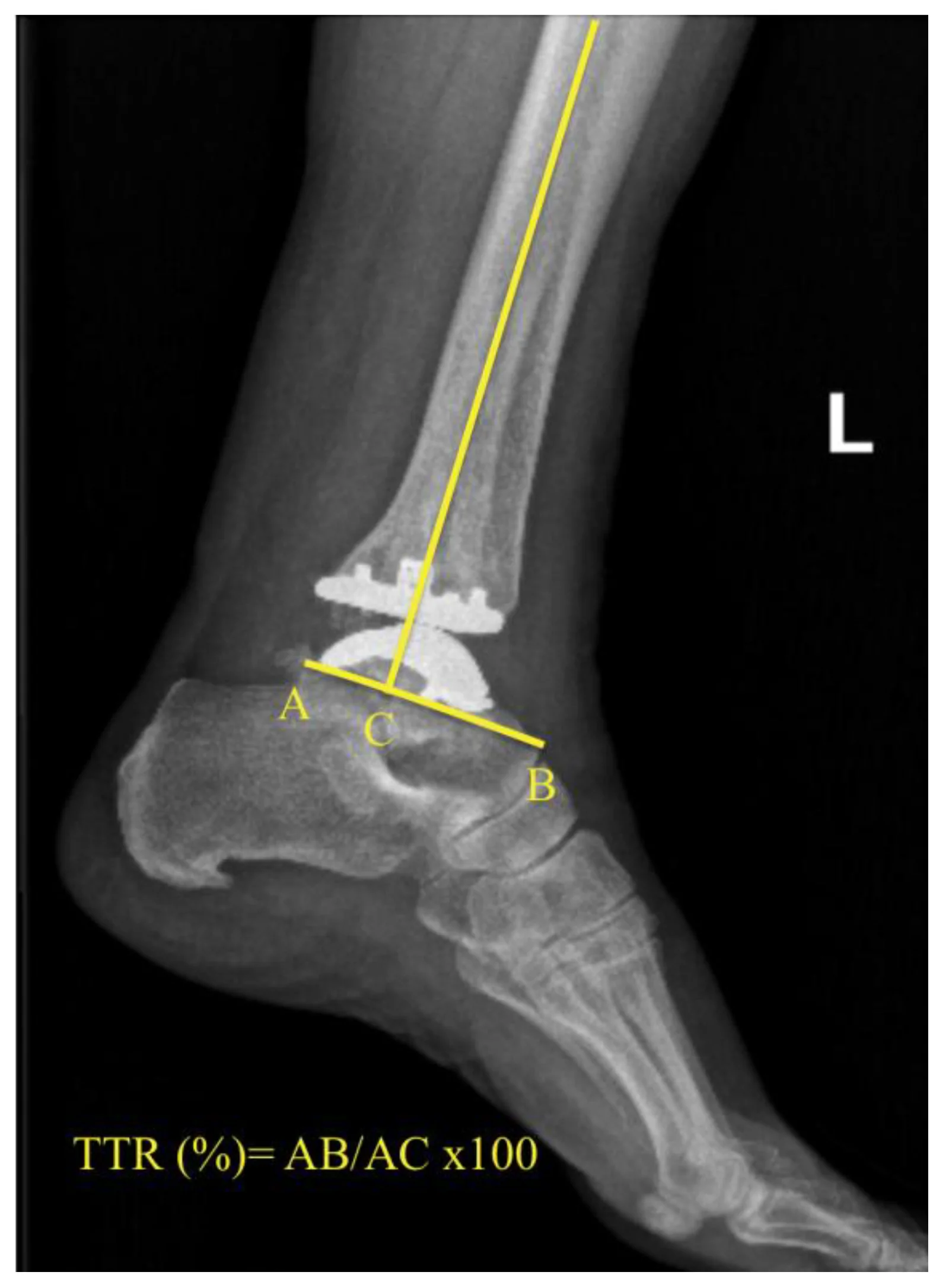
Tibiotalar ratio (TTR): evaluates the anteroposterior position of the talus relative to the tibia. It is measured along the talar longitudinal axis (TALAX), using three reference points: Point A: posterior aspect of the talar component. Point B: intersection between TAX and TALAX. Point C: intersection between TALAX and the talar head. The TTR is calculated as the ratio AB/AC, expressed as a percentage.

**Table 1:** Radiographic parameters for the evaluation of tibial component in TAR, including optimal, acceptable and malpositioned values.

Parameter	Optimal Value	Acceptable Value	Malpositioning
Tibial coronal angle (α angle)	90°	87–93°	>3–5° deviation
Tibial sagittal angle (β angle)	90°	87–93°	>3° deviation
Talar sagittal angle (γ angle)	17–19°	10–25°	Over-dorsiflexion >25–30° Over-plantarflexion <10° A change of >5° over time indicates implant migration or loosening
Tibiotalar ratio (TTR)	27–42%	27–42%	<27% and >42%
AP offset ratio	0	−0.05–+0.05	>0.05 anterior translation of talar component <0.05 posterior translation of talar component
Radiolucent lines	Absent	≤2 mm, non progressive	>2 mm or progressive
Subsidence	Absent	≤5 mm	>5 mm

## Data Availability

The data are available on request to the corresponding author.

## Abbreviations

The following abbreviations are used in this manuscript:

aSDTA	Anatomic sagittal distal tibial angle
OA	Ankle osteoarthritis
AP	Anteroposterior
AP Offset Ratio	Anteroposterior offset ratio
AOFAS score	American Orthopaedic Foot and Ankle Society score
CAX	Anatomical axis of the calcaneus
CT	Computed tomography
FB	Fixed-bearing
HFA	Hindfoot alignment angle
MDTA	Medial distal tibial angle
MB	Mobile-bearing
TALAX	Talar longitudinal axis
TAX	Tibial anatomical axis
TTR	Tibiotalar ratio
TSA	Tibiotalar surface angle
TAS	Tibial anterior surface angle
TAR	Total ankle replacement
UHMWPE	Ultra-high-molecular-weight polyethylene
VAS	Visual analogue scale
WBCT	Weightbearing computed tomography
WBXR	Weightbearing radiographs
